# Revisions after prior stapes surgery: aspects on indication, intraoperative findings and surgical strategies

**DOI:** 10.1007/s00405-024-09035-8

**Published:** 2024-11-26

**Authors:** Kariem Sharaf, Ivo Grueninger, Sara Alekuzei, Daniel Polterauer, Andrea Schreier, Martin Canis, Tobias Rader, John Martin Hempel, Joachim Müller

**Affiliations:** 1 Department of Otorhinolaryngology, University Hospital, LMU Munich, Marchioninistrasse 15, 81377 Munich, Germany; 2 Department of Otorhinolaryngology, Helios Hospital Berlin-Buch, Schwanebecker Chaussee 50, 13125 Berlin, Germany

**Keywords:** Stapes revision surgery, Stapedectomy, Otosclerosis, Complications, Surgical outcome

## Abstract

**Objective:**

Primary stapes surgery is considered a challenging intervention in ear surgery. Despite an risk of deafness in 0.5–1 percent, this procedure has still a good benefit-risk ratio due to the improvement in hearing and quality of life that is usually achieved. However, revision after prior stapes surgery is considered even more challenging. Revisions after stapes surgery are very heterogeneous procedures, both in terms of the indication and the surgical strategy and are generally considered to be significantly more demanding. Reasons for complications after prior stapes surgery as well as strategies for successful revisions are not well described in the literature.

**Methods:**

Retrospective cohort study, tertiary referral center**.** 124 cases of revisions after prior stapes surgery were identified between 2011—2022 and are analyzed based on biographic data, clinical, audiological, and intraoperative findings as well as the eventual therapy. Cases were analyzed regarding indication, intraoperative finding and the surgical strategy chosen.

**Results:**

Acute, subacute, and long-term complications of the primary intervention as well as other incidental reasons such as progressive hearing loss can be identified as indication for revision surgery. Preoperative clinical findings were correlated to intraoperative findings and surgical strategies. Audiological results are discussed.

**Conclusions:**

Different recommendations for the indication of a surgical revision can be derived depending on the individual preoperative case history and findings. In addition, there are patterns regarding the chances of success of a revision, especially in cases of persistent conductive hearing loss chances of hearing improvement seem possible in more than 80% of cases.

## Introduction

Otosclerosis is a disease of the otic and labyrinthine capsules [1]. It leads to a progressive, predominantly conductive hearing loss due to bony fixation of the stapes footplate to the oval window, accompanied by typical symptoms such as tinnitus [2–4]. Stapes surgery is an effective treatment of hearing loss and tinnitus in most cases, both as a primary option of therapy and in case of revision surgery [5–7]. Moreover, the method leads to improved quality of life [8].

As John Shea recounted in a personal communication with the last author, his first stapes surgeries performed on 14.9.1955 and 1.5.1956 were initially discussed extremely critically in professional circles due to the experiences of Johannes Kessel. Kessel had to experience partly serious complications during his stapes surgeries, which in the meantime had also led to the abandonment of the method. Initiated by the surgical technique introduced by John Shea, several innovations and detailed improvements were made over the decades [9, 10]. Early custom-made wire connective tissue prostheses were abandoned in favor of piston prostheses made of a wide variety of materials [11]. It seems important to note that a larger piston diameter of 0.6 mm leads to better postoperative results [5, 12]. Other factors that may influence the outcome include the material and design of the prostheses, details of their fixation mechanism, the surgical technique (for example, stapedectomy vs. stapedotomy, laser vs. manual perforation vs. slow-rotating microdrills, use of endoscopic techniques) as well as the influence of the surgeon are discussed [6, 11, 13–21].

Due to that, primary stapes surgery with stapedotomy or stapedectomy is considered a very challenging intervention in ear surgery, including an acute risk of deafness estimated as 0.5–1 percent [8, 22]. The risk and benefit of stapes revision surgery are discussed controversially in the literature, and the risk of deafness after stapes revision surgery is estimated to be up to 10 times higher than after primary stapedectomy [18, 23, 24]. Revision cases depict very heterogeneous procedures, both in terms of the indication, intraoperative findings, and the surgical strategy. The intraoperative findings in stapes revision surgery are diverse and difficult to predict in individual cases. This article tries to associate preoperative symptoms that lead to the indication of stapes revision surgery with intraoperative findings and surgical strategies. Thereby, we try to categorize different variants of stapes revision surgery and look for predictors for the audiological outcome. Further, we try to gain an understanding whether complications after stapes surgery are prosthesis- or procedure-related and in which cases a revision can be beneficial for the individual patient. This information might help otologists counseling their patients and planning revision stapes surgery.

## Material and methods

### Case selection

For this explorative retrospective single-center study, 124 cases of stapes revision surgery were identified. All surgeries were performed between March 2011 and December 2022. Potential cases were identified by searching for International Classification of Diseases version 10 (ICD-10) and “OPS codes” (“*Operationen- und Prozedurenschlüssel*”, roughly translated: Operation and Procedure Classification System, a German modification of the International Classification of Procedures in Medicine) for stapes surgery, revision stapes surgery, and, in some cases, cochlear implantation when ICD-10 coding indicated the presence of a pre-existing prosthesis in the clinical SAP ® database of our clinic. More than 500 cases were reviewed and, subsequently, a majority of cases were excluded. Reasons for exclusion included any primary stapes procedures and any surgical procedures when there were no signs of pre-existing stapes surgery.

### Data collection

Basic patient characteristics, anamnestic and clinical data on preoperative symptoms as well as surgery-related details and audiometric measures of the surgical cases were obtained. To evaluate all reasons for revision, we also included cases with missing audiometric data. Since our data was collected at a tertiary referral center, not all revision surgeries underwent primary surgery at our institution. Therefore, data on the exact date of prior surgery, preoperative audiometry as well as details on the primary surgical procedure including type of prosthesis was incomplete. In some cases, primary surgery was performed years ago, and the exact date of primary surgery was unknown. In these cases, the date for prior stapes surgery was set to mid-month or mid-year. If the year of the prior surgery was unknown, the case was excluded for analysis of time to revision. Primary outcome measures were indication for revision, intraoperative findings as well as surgical strategy. Secondary outcome measures were audiometric data. We collected data for intraoperative findings as well as the surgical strategy from surgical reports that were not standardized. Although revisions were performed by different surgeons within our center, the same standards and techniques were used.

### Classification of indication of revision

Heterogenous reasons for revisions were found and sometimes multiple symptoms led to revision. If more than one symptom led to revision, the leading symptom was chosen as an indication for revision based on the documentation of in-house counseling. Nevertheless, any indication that led to revision was recorded. After revision of all cases, we defined categories to 1. conductive hearing loss that was persistent after prior stapes surgery (CHLpers), 2. conductive hearing loss that re-occurred after prior stapes surgery (CHL_new_), 3. sensorineural hearing loss that occurred acutely after prior stapes surgery (SNHL_acute_), 4. sensorineural hearing loss that was progredient after prior stapes surgery (SNHL_prog_), 5. vertigo, 6. tension phenomena (including symptoms such as change of hearing thresholds after Valsalva, rattling or distortion for certain frequencies or loud noises), 7. bleeding after surgery, 8, chronic otitis media (OMC) after stapes surgery, 9 tinnitus and 10. facial palsy.

### Classification of intraoperative finding

In many cases, multiple intraoperative findings were documented in the surgical report. All findings were extracted. Intraoperative findings that were observed are 1. erosion of ossicular chain (incus and malleus), 2. dislocation of prosthesis (separated for dislocation into vestibulum and tympanum), 3. loosening of prosthesis, 4. scar formation, 5. adhesion of tympanic membrane, 6. fixation of ossicular chain, 7. fixation of stapes prosthesis, 8. (re-)obliterated stapedotomy, 9. extrusion of the stapes prosthesis, 10. granuloma / toxic process, 11. perilymphatic fistula, 12. cholesteatoma and 13. cases with no intraoperative findings.

### Classification of surgical strategies

Surgical strategies were recorded based on the surgical reports. If more than one procedure was documented all procedures were recorded. Procedures were: 1. (re)-fixation of prosthesis, 2. replacement of prosthesis, 3. (re)-stapedectomy, 4. obliteration of oval window, 5. tympanoplasty, 6. scar disintegration, 7. disintegration of fixations of ossicular chain, 8. malleovestibulopexy, 9. hemostasis, 10 explantation of the stapes prosthesis, 11. cochlear implantation and 12. active middle ear implantation (AMEI).

### Classification of prosthesis- and procedure-related complications

To the best of our knowledge, there is no clear agreement on the classification of complications after stapes surgery to prosthesis- or procedure-related. In this study, we classified ossicular chain erosion, dislocation of prosthesis, and loosening of prosthesis as prosthesis-related complications. Scar formation, adhesion of tympanic membrane, isolated (re-)obliterated stapedectomy (maybe due to faulty stapedectomy or too short stapes prosthesis), perilymphatic fistula, facial palsy, bleeding, OMC as well as cholesteatoma after stapes surgery were classified as procedure-related complications. Granuloma / toxic process, (maybe priorly unidentified) fixation of ossicular chain and cases with no clear intraoperative findings were not counted in either category since it seems unclear whether the cause of this complication is prosthesis- or procedure-related. Sensorineural hearing loss that was progredient after prior stapes surgery and led to revision was not accounted to prosthesis- nor procedure-related since progredient SNHL is commonly caused by otosclerosis itself or presbycusis. In cases where several findings were described (e.g. dislocation of prosthesis and closed stapedectomy), the leading failure mode as identified and described by the surgeon who performed revision was counted.

### Classification of time-to-revision

Regarding heterogenous indications for stapes revision surgery, the duration of prior stapes surgery to revision seems to be relevant in terms of patterns of complications that lead to revision and findings that are expected. To the best of our knowledge, there is no standardized classification for the timing of stapes revision. Therefore, we aim to classify revisions based on the duration since prior stapes surgery. For our cohort, we identified three major groups: early revisions within 12 months further subdivided into acute revisions within 1 month and subacute revisions within 1 month – 12 months, intermediate revisions within 12 months—5a, and late with revision > 5a after prior stapes surgery.

### Audiometric measurements

For further evaluation of audiometric data, if accessible, data was transferred to a science-related surgical Otolaryngology clinical database (ENTstatistics, INNOFORCE, Ruggell, Liechtenstein). Pure tone audiometry data was collected preoperatively and postoperatively. Results were obtained at frequencies 0.125, 0.25, 0.5, 0.75, 1, 1.5, 2, 3, 4, 6, 8, and 10 kHz for bone conduction (BC) and air conduction (AC), if available.

Audiometry testing was performed using an AT1000 audiometer (Auritec Medizindiagnostische Systeme, Hamburg, Germany) with DT48A headphones (Beyerdynamic, Heilbronn, Germany) and a B71 bone conduction vibrator (Radioear, Middelfart, Denmark). The measurements were collected using standardized audiometric procedures in compliance with ISO norm 8253–1:2010 and reported according to the guidelines of the Committee on Hearing and Equilibrium [25, 26].

The air–bone gap (ABG) was calculated using pure tone averages (PTA) of the commonly obtained frequencies 0.5, 1, 2, and 3 kHz. In few cases, audiometric data for 3 kHz was missing and was therefore calculated by averaging the measured thresholds at 2 and 4 kHz as described previously. [27] Hearing improvement (HI) is defined as the difference of preoperative minus postoperative AC from PTA. ABG closure is defined as the difference of preoperative minus postoperative ABG from PTA. Overclosure (or perioperative inner ear damage) is detected when subtracting postoperative from preoperative BC (PTA). Additional audiological outcome measures of this study were relative HI and relative ABG closure as described before [7]. Relative HI is defined as the quotient of HI divided by the preoperative ABG from PTA. Relative ABG closure is defined as the quotient of ABG closure divided by the preoperative ABG from PTA. Formulas for the aforementioned measures are shown below [7]:

$$HI=preoperative {AC}_{PTA}- postoperative {AC}_{PTA}$$

$$ABG closure=preoperative\,{ABG}_{PTA}-postoperative {ABG}_{PTA}=\left(preoperative {AC}_{PTA}-preoperative {BC}_{PTA}\right)- \left(postoperative {AC}_{PTA}-postoperative {BC}_{PTA}\right)$$

$$Overclosure=preoperative\,{BC}_{PTA}-postoperative\,{BC}_{PTA}$$

$$relative\,HI=\frac{HI}{preoperative\,{ABG}_{PTA}}$$

$$relative\,ABG\,closure=\frac{ABG\,closure}{preoperative\,{ABG}_{PTA}}$$

### Statistical analysis

Statistical analysis was performed using Excel (Microsoft, Redmond, WA) and OriginPro 2021 (OriginLab Corp., Northampton, MA). All data were tested for normality using the Shapiro–Wilk test. For comparison between paired groups, the Wilcoxon signed rank test, and for comparison between unpaired groups, the Mann–Whitney-U test was used. A value of p < 0.05 was considered significant. To meet our primary outcome measure heatmaps of indication and correlating intraoperative findings as well as indication and chosen surgical strategy were plotted.

## Results

Detailed baseline characteristics, number of revisions of the selected cases, intraoperative findings, and audiometric measures, including preoperative and postoperative ABG and relative hearing outcome measures are shown in Table 1. 69/124 cases were primary revisions while 52/124 cases received secondary or more revisions. Number of revision was unknown for 3 cases. For cases that received at least re-revision, 24/52 cases had prior revision at our center while 28/52 cases had prior revision elsewhere.

**Table 1:** Case characteristics

Characteristics		Surgeries performed (n = 124)
Patients [n]		98
Sex [%]	Male Female	31.63 68.37
Mean age at surgery [years] ± SD		52.35 ± 15.07
Revision of the ear [n]	Primary Secondary Tertiary Quartary Quintary Unknown	69 33 15 3 1 3
Prior surgery at our center		48 (38.7%)

### Indication of revision

Sometimes we found multiple indications that led to revision. Distribution of indication for revision was: CHL_new_: 60/124 (48,8%), SNHL_acute_: 30/124 (24,2%_)_, CHL_pers_: 27/124 (21,8%), vertigo: 27/124 (21,8%), SNHL_prog_: 25/124 (20,2%), tinnitus: 23/124 (18,5%), tension phenomena: 17/124 (13,7%)., OMC: 7/124 (5,6%), bleeding after surgery: 3/124 (2,4%), and facial palsy: 1/124 (0.8%). Most common combinations of indications were SNHL_acute_ + vertigo (17/124) and SNHL_prog_ + CHL_new_ (12/124) followed by the combination of CHL_new_ + tension phenomena, CHL_new_ + vertigo, and CHL_pers_ + tinnitus (each 9/124).

### Time to revision

We observed a broad spectrum of time intervals between prior surgery and revision surgery with a median of 54.4 months (minimum 0 days (within 24 h after initial surgery); maximum of 45.1 years; Q1 5.9 months; Q3 172.4 months) in 118 cases. For 6 other cases, the date of the prior surgery was unknown.

To classify time to revision we defined 3 major groups: early revisions within 12 months to earlier stapes surgery (group I), intermediate revisions within 1 – 5 years after prior stapes surgery (group II), and late revisions after more than 5 years (group III). Taking our observations into account, we further subdivided early revisions into acute revisions within 1 month (group Ia) and subacute revisions between 1 and 12 months (group Ib).

In our cohort, we found 39/124 (31.5%) early revisions (group I) further divided into 17/124 (13.7%) acute revisions (group Ia) and 22/124 (17.7%) subacute revisions (group Ib). 23/124(18.5%) were intermediate revisions (group II) while 56/124 (45.2%) were late revisions (group III). 6/124 (4.8%) were unknown.

When separating time to revision according to indication for revision, we found major differences between indications: earliest were bleeding (n = 3, all within 3 d), facial palsy (one case with revision after 42 d), vertigo (n = 26, med 61 d, Q1 5d, Q3 121.3 mo) and SNHL_acute_ (n = 29, med 90d, Q1 7d, Q3 59.4 mo) followed by CHL_pers_ (n = 27, med 13.0 mo, Q1 189 d, Q3 48.3 mo), tension phenomena (n = 17, med 28.8 mo, Q1 19.0 mo, Q3 120.0 mo), tinnitus (n = 23, med 49.7 mo, Q1 133d, Q3 159.5 mo), OMC (n = 7, med 57.6 mo, Q1 40.7 mo, Q3 148.1 mo), CHL_new_ (n = 58, med 127.4 mo, Q1 57.6mo, Q3 202.1 mo) and SNHL_prog_ (n = 22, med 267.6 mo; Q1 139.4 mo; Q3 344 mo).

**Fig. 1:**
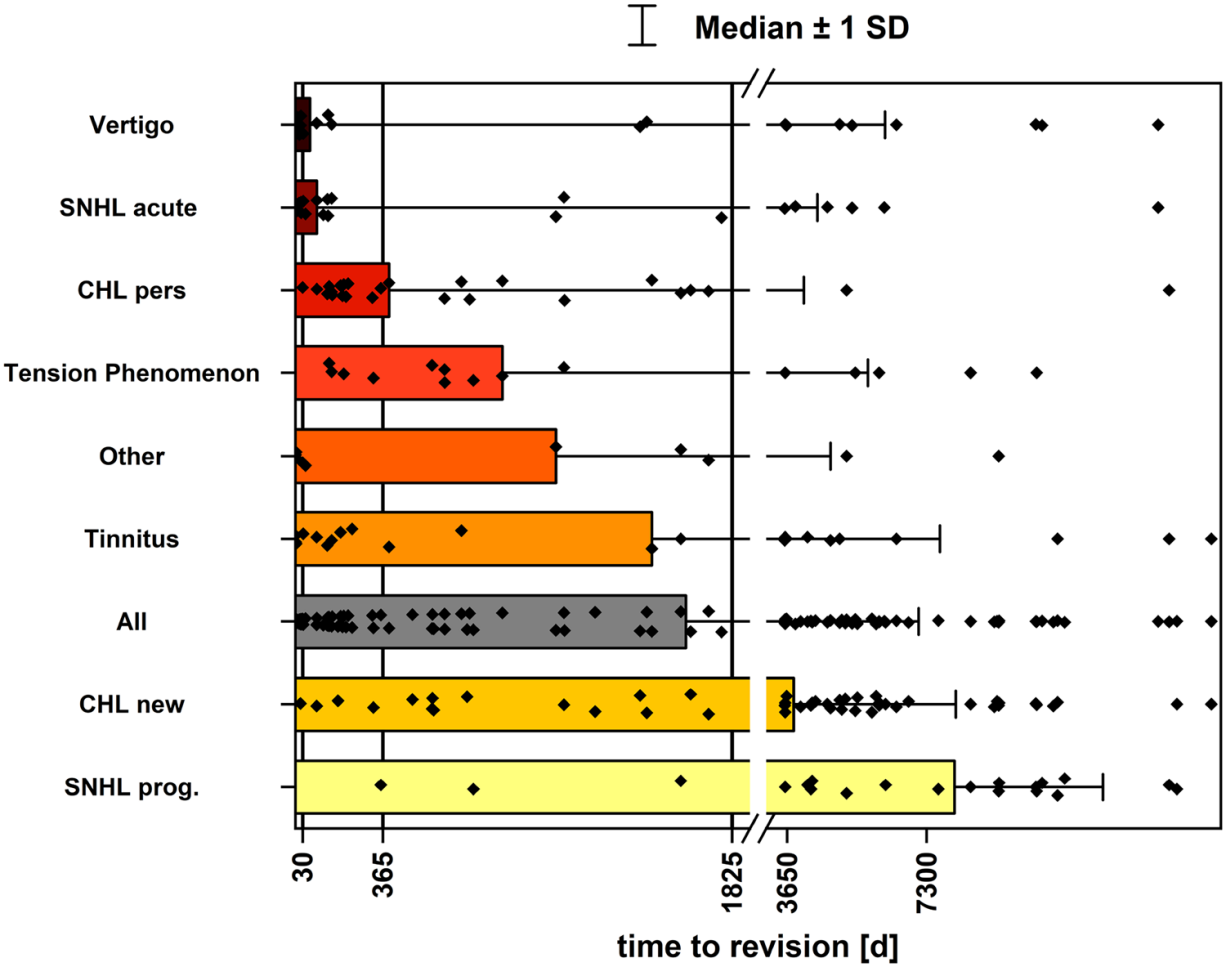
Time since last stapes surgery depending on the preoperative findings

Time to revision is depicted in Fig. 1 with indications bleeding, facial palsy, and OMC summed up as “others”.

Leading indications for acute revisions (group Ia) were bleeding (3/17), SNHL_acute_ (12/17) mostly in combination with vertigo (8/12) or vertigo and tinnitus (3/12), isolated vertigo (1/17) and OMC with CHL_pers_ (1/17). Subacute revisions (group Ib) were mainly caused by CHL_pers_ (10/22) (standing-alone 6/10, accompanied with tinnitus 2/10, tension phenomena 1/10 or SNHL_prog_ 1/10); SNHL_acute_ (7/22) accompanied by vertigo and/or tinnitus (3/7), CHL_pers_ (2/7) or facial palsy (1/7); CHL_new_ (3/22) (accompanied with vertigo and tinnitus 1/3 or tension phenomena 1/3); tinnitus (1/22) or tension phenomena (1/22).

Intermediate revisions (group II) were performed due to CHL_new_ (10/23) sometimes in combination with vertigo (2/10) or OMC (1/10); CHL_pers_ (8/23) in times accompanied by tinnitus (3/8) or tension phenomena (2/8); SNHL_acute_ (3/23) sometimes in combination with OMC (1/3) or CHL_new_ and tension phenomena (1/3); OMC with SNHL_prog_ (1/23) and stand-alone tension phenomena (1/23).

Indications for late revisions were mainly CHL_new_ 40/56 (stand-alone 21/40, in combinations with: SNHL_prog_ 6/40, tinnitus 4/40, SNHL_prog_ + tension phenomena 3/40, tinnitus + vertigo 2/40, tension phenomena + vertigo + SNHL_prog_ (1/40), vertigo 1/40,, OMC + SNHL_prog_ (1/40), SNHL_prog_ + vertigo (1/40). Other indications for late revision were SNHL_acute_ 7/56 (with vertigo 2/7, with CHL_new_ 2/7 and with CHL_new_ + Tinnitus 1/7), SNHL_prog_ 7/56 (in combination with CHL_pers_ and tinnitus 2/7, vertigo 1/7, CHL_pers_ + OMC 1/7) as well as OMC (1/56) and the combination of CHL_pers_ + tension phenomena + tinnitus (1/56).

### Intraoperative findings

Most common intraoperative findings were scar formations (73/124), incus erosion (49/124), dislocation of the prosthesis (49/124 with 17/49 dislocation into the vestibulum and 32/49 dislocation into the tympanum), loosening of the prosthesis (34/124), (re-)obliterated stapedoctomy (26/124), granuloma / toxic process (22/124) and perilymphatic fistula (13/124). Less common findings were adhesion of tympanic membrane (8/124), fixation of stapes prosthesis (7/124), cholesteatoma (6/124), fixation of ossicular chain (5/124), erosion of malleus (3/124), extrusion of the stapes prosthesis through the tympanic membrane (3/124) and cases with no intraoperative findings (7/124).

**Fig. 2:**
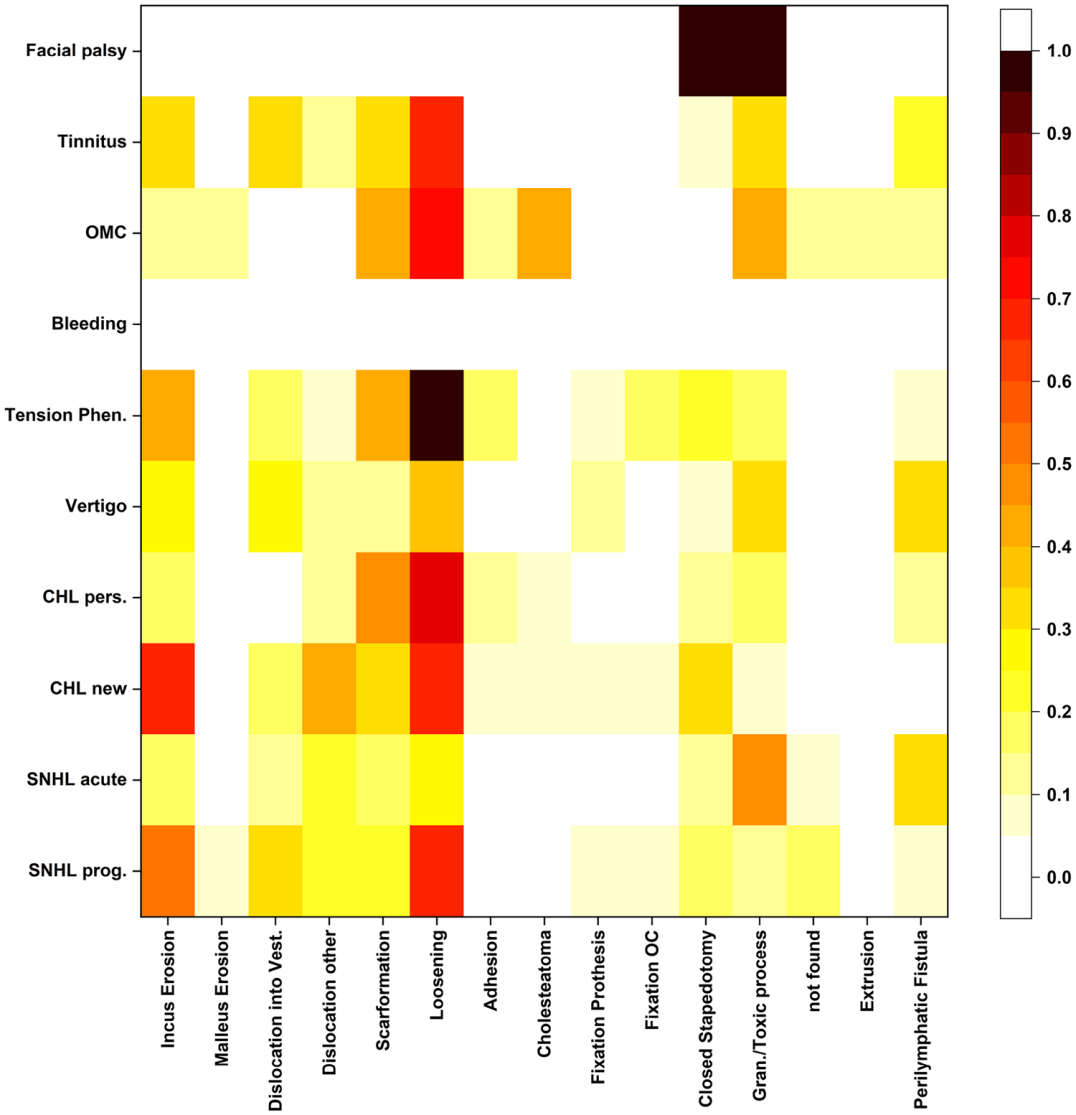
Correlation of preoperative and intraoperative findings in stapes revision surgery: Fields in red/darker fields depict higher correlations between a preoperative and an intraoperative finding, black indicates a correlation of 1.0 (equals 100%). There was only one case with preoperative facial palsy, which was referred to us after external stapes revision surgery, explaining the extremely high and low correlations

To correlate the intraoperative finding with the symptoms that led to indication for revision we plotted a heatmap with the correlations between findings and indications as depicted in Fig. 2.

The most common indication for revisions was CHL_new_. In these cases, common intraoperative findings were incus erosion (66.7%), scar formation (68.3%), dislocations (58.3% with 40% into the tympanum and 18.3% into the vestibulum), loosening as well as (re-)obliterated stapedotomy (both 31,7%). The second most common indication was SNHL_acute_ and most typical findings for these cases were granuloma / toxic process (46.7%) followed by perilymphatic fistula (33.3%) and dislocations (33.3%) with dislocation into vestibulum in 13.3% and into the tympanum in 20% of cases. For cases revised due to CHL_pers_ after prior stapes surgery typical findings were scar formation (77.8%) and loosening of the prosthesis (48.1%). The most typical findings for cases with indication tension phenomena were loosening and incus erosion (both 41.2%). In cases indicated because of vertigo, typical intraoperative findings were granuloma / toxic process and perilymphatic fistulas (both 33.3%) as well as dislocations of prosthesis into the vestibulum (25.9%) next to incus erosion (29.6%) and scar formation (37.0%). In cases that presented with tinnitus frequently reported intraoperative findings were scar formation (65.2%), dislocation (all: 43.5%, into the vestibulum: 30.4%), incus erosion (34.8%) as well as loosening and granuloma / toxic process (both 30.4%). In cases revised because of SNHL_prog_, regular intraoperative findings were scar formations (68.0%) as well as incus erosion (52.0%).

### Surgical strategies

The most frequently performed surgical solution via endaural approach was the replacement of the stapes prosthesis (53.2% of all cases). Replacement was especially frequent for cases that presented with CHL_new_ (78.3% received a new prosthesis) and tension phenomena (76.5%). Also, scar disintegration was a regularly described procedure (45.2% of all cases) and was most common in cases with tension phenomena (82.4%), CHL_pers_ (70.4%), OMC (71.4%), and tinnitus (60.9%). Obliteration of the oval window (OW) was regularly performed (25.0% of all cases) most commonly in cases that presented with SNHL_acute_ (OW-obliteration performed in 56.7% of these cases) and vertigo (55.6%). In cases with SNHL_acute_, the existing prosthesis was explanted in 33.3%, for cases with vertigo explantation was performed in 29.6% (explantation rate for all cases was 9.7%). Cochlear implantation (CI) was performed in 19/124 cases (15.3%), especially for indication SNHL_prog_ (32.0% of all such cases received a CI) and SNHL_acute_ (26.7%).

Less common procedures were re-fixation of the priorly implanted prosthesis (6.5% of all cases), tympanoplasty (12.9%), malleovestibulopexy (2 cases, 1.6%), (re)-stapedectomy (4.8%), AMEI (2.4%), hemostasis (4.0%) and disintegration of fixations (7.2%).

**Fig. 3:**
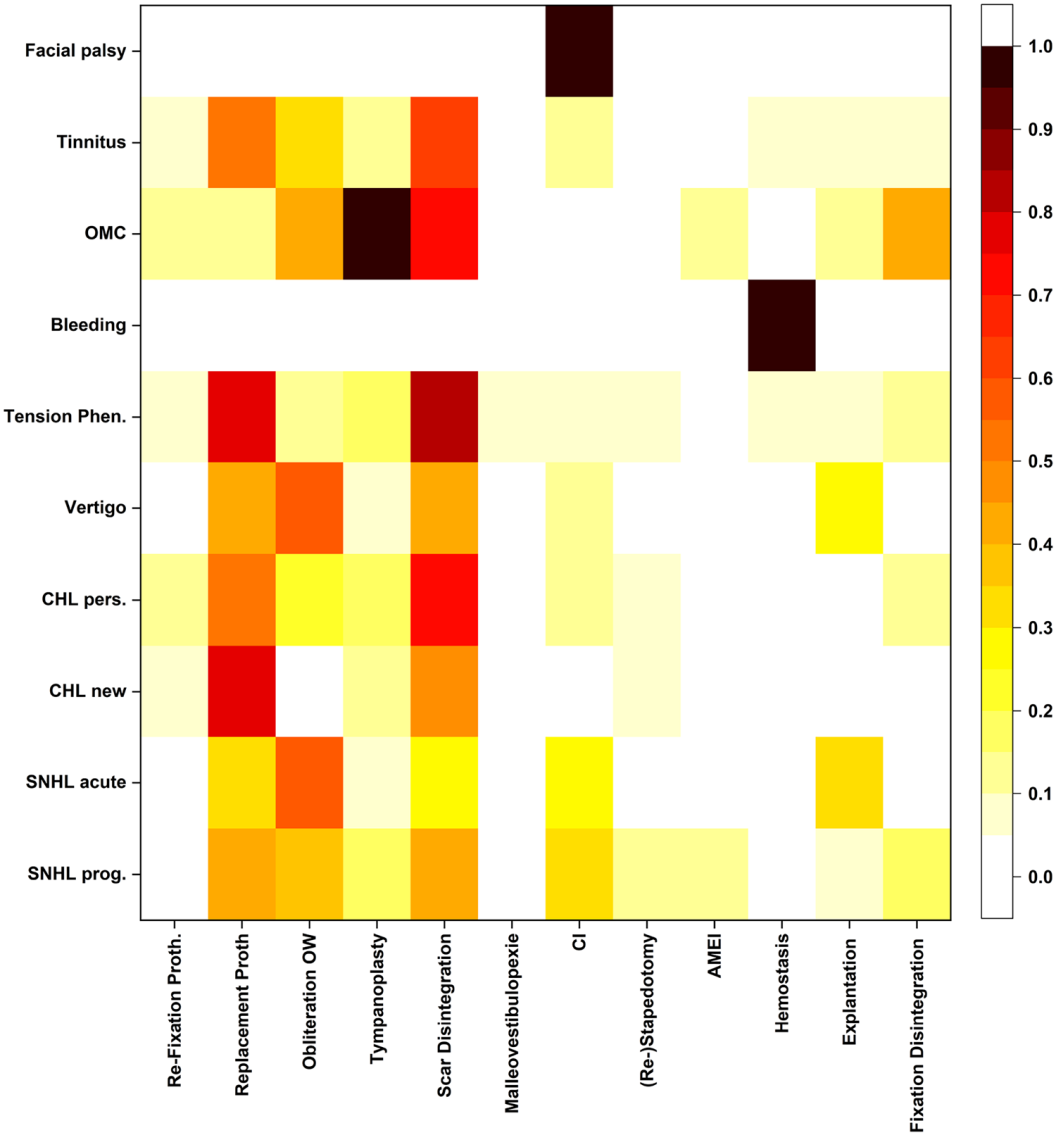
Correlation of preoperative findings and surgical solutions in stapes revision surgery: Fields in red/darker fields depict higher correlations between a preoperative finding and an intraoperative solution used. Black indicates a correlation of 1.0 (equals 100%). There was only one case with preoperative facial palsy, which was referred to us after external stapes revision surgery, and three cases with bleeding explaining the extremely high and low correlations

Figure 3 shows a heatmap with the correlations between surgical strategy and indication.

### Classification of prosthesis- and procedure-related complications

In our cohort, we classified 66/124 (53.2%) of the observed failure modes / complications as prosthesis-incus interface related (ossicular chain erosion, dislocation of prosthesis and / or loosening of prosthesis). In 26/124 (21.0%) cases, the failure mode / complication was classified as procedure-related (scar formation as main failure mode, adhesion of tympanic membrane, isolated (re-)obliterated stapedectomy, perilymphatic fistula, facial palsy, bleeding, OMC, cholesteatoma). In 32/124 (25.8%) cases it was not possible to clearly identify or classify the failure mode as prosthesis- or procedure-related.

### Audiometric measurements

**Fig. 4:**
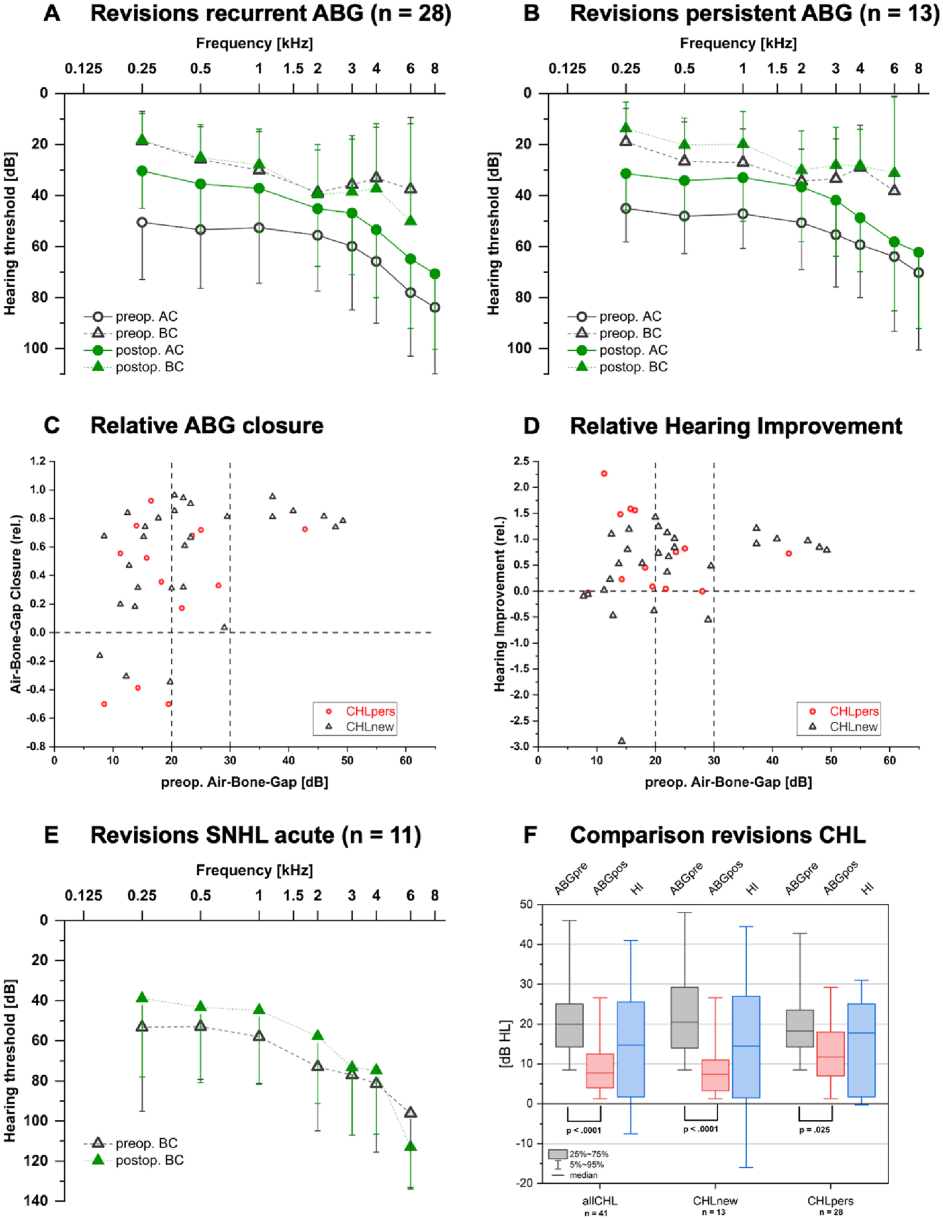
Audiometric outcomes. **A**–**D** Audiometric outcome of cases with recurrent (**A**, black triangles in **C** and **D**) or persistent (**B**, red dots in **C** and **D**) conductive hearing loss (air bone gap, ABG); **E** Cumulative audiogram of acute revision cases depicting bone conduction audiometry pre- and postoperatively; **F** Comparison of audiometric outcome of cases with conductive hearing loss (left columns) and subgroups recurrent (middle columns) or persistent (right columns) conductive hearing loss, Tables “Revisions after prior stapes surgery: Aspects on indication, intraoperative findings and surgical strategies”

To relate to our secondary outcome parameter, we analyzed audiological results wherever data was accessible. For many revision cases, we did not find usable preoperative audiological data either because the case was referred to our center after prior stapes surgery elsewhere or because of acute revision without the possibility of exact audiological assessment preoperatively. All in all, we were able to find usable audiological data sets for 57 cases (listed by indication for revision: CHL_new_ n = 28; CHL_pers_ n = 13; SNHL_acute_ n = 11; SNHL_prog_ n = 3; OMC n = 2). Because of the small sample size, indications OMC and SNHL_prog_ were excluded for further analysis. The remaining 52 cases were analyzed as described above. For all cases of the indication groups with CHL in general, CHL_new_, and CHL_pers_, the outcome parameters relative ABG closure and relative HI, the commonly used outcome measures including ABG and ABG closure as well as absolute hearing improvement are listed in Table 2. Regarding all revision cases indicated because of CHL, the ABG improved significantly after revision (p < 0.0001, Wilcoxon signed rank test, Fig. 4F). Also, when separating the groups for CHL_new_ and CHL_pers_ the ABG improved significantly for both groups (p < 0.0001 for CHL_new_, p = 0.025 for CHL_pers_, both Wilcoxon signed rank test, Fig. 4F). There was no significant difference in HI between the subcohorts CHL_new_ and CHL_pers_ (p = 0.96, Mann–Whitney-U-test). Figure 4 A, B and E depict cumulative pure tone audiograms of the SNHL_acute_ and CHL cohorts. Figure 4 C and D displays both outcome parameters relative ABG closure and relative HI in dependence of the type of indication for revision and the preoperative ABG in the CHL cohorts.

**Table 2:** Audiometric outcomes of conductive hearing loss cases

	CHL all	CHL_new_	CHL_pers_
Number of cases n	41	28	13
ABG preoperative median (min, max, Q1, Q3)	20.0 dB (min 7.75 dB, max 49.25 dB, Q1 14.25 dB, Q3 25.0 dB)	20.5 dB (min 7.75 dB, max 49.25 dB, Q1 14.0 dB, Q3 29.25 dB)	18.25 dB (min 8.5 dB, max 42.75 dB, Q1 14.25 dB, Q3 23.5 dB)
ABG postoperative median (min, max, Q1, Q3)	7.75 dB (min 0.75 dB, max 29.25 dB, Q 4.0 dB, Q3 12.5 dB)	7.38 dB (min 0.75 dB, max 28.0 dB, Q1 3.25 dB, Q3 10.96 dB)	11.75 dB (min 1.25 dB, max 29.25 dB, Q1 7.0 dB, Q3 18.0 dB)
ABG closure median (min, max, Q1, Q3)	10.5 dB (min − 9.75 dB, max 38.6 dB, Q1 4.5 dB, Q3 19.75 dB)	12.54 dB (min − 6.83 dB, max 38.6 dB, Q1 5.13 dB, Q3 22.5 dB)	8.25 dB (min − 9.75 dB, max 31.0 dB, Q1 3.75 dB, Q3 15.25 dB)
ABG closure relative median (min, max, Q1, Q3)	0.67 (min − 0.5, max 0.96, Q1 0.31, Q3 0.81)	0.71 (min − 0.35, max 0.96, Q1 0.31, Q3 0.83)	0.52 (min − 0.5, max 0.92, Q1 0.17, Q3 0.72)
HI median (min, max, Q1, Q3)	14.75 dB (min − 41.25 dB, max 45.0 dB, Q1 1.75 dB, Q3 25.5 dB)	14.5 dB (min − 41.25 dB, max 45.0 dB, Q1 1.5 dB, Q3 27.0 dB)	17.75 dB (min − 0.25 dB, max 31.0 dB, Q1 1.75 dB, Q3 25.0 dB)
HI relative median (min, max, Q1, Q3)	0.73 (min − 2.89, max 2.27, Q1 0.09, Q3 1.01)	0.76 (min − 2.89, max 1.43, Q1 0.12, Q3 1.01)	0.73 (min − 0.03, max 2.27, Q1 0.09, Q3 1.48)

Importantly, we observed 2 cases of major deterioration of BC or deafness after revision surgery for CHL. Both cases had CHLnew as the leading indication for revision surgery and intraoperatively presented with dislocation of the prosthesis in both cases (into vestibulum for one case and other for the second case). Both cases received 2 more revisions afterwards: case 1 with obliteration of the OW because of perilymphatic fistula and eventually CI because of total deafness after 2nd revision; case 2 with replacement of the prothesis because of reobliterated OW and eventually CI after stable functional deafness.

For the indication group SNHL_acute_ we only recorded the bone conduction levels pre- and postoperatively. For these cases the median BC preoperative (PTA) was 67.5 dBHL (min 35.75 dBHL, max 90.75 dBHL, Q1 51.38 dBHL, Q3 79.63 dBHL) and the median BC postoperative (PTA) was 44.75 dBHL (min 17.5 dBHL, max 120 dBHL, Q1 34.92 dBHL, Q3 53.38 dBHL). The BC (PTA) did not change significantly after revision (p = 0.27, Wilcoxon signed rank test). In our cohort, 8/11 cases with indication SNHL_acute_ had an improved BC (PTA) after revision while 2/11 cases had a worse BC (PTA) and 1/11 cases had no relevant change of BC (PTA).

The 2 cases with worse BC (PTA) after revision for SNHL_acute_ are further described. Case 1 had revision because of major acute SNHL after prior stapes surgery 10a before. Intraoperatively dislocation of the prosthesis into the vestibulum was observed. The patient received a new prosthesis and postoperative BC showed a complete deafness. Case 2 represents one of the patients described above with major deterioration of BC close to deafness after revision stapes surgery for CHL_new_. The patient was revised for SNHL_acute_ and a perilymphatic fistula was observed and obliteration of the OW was performed. Postoperatively BC presented deafness and the patient received a CI afterwards.

## Discussion

Facing a great variety of indications, findings and surgical strategies, the main goal of the study was to explore options to categorize cases of revision surgery after prior stapes surgery. Conclusions regarding this primary goal were mainly based on preoperative and intraoperative findings.

Regarding the time between the latest stapes or middle ear surgery or onset of symptoms, our analysis directs us in a categorization of three types of stapes revision surgery: I. an acute type Ia hours to 30 days and subacute type Ib 30 days to 12 months after the latest stapes or middle ear surgery usually after onset of acute severe symptoms such as severe, objectively verifiable vertigo or severe acute hearing loss/deafness which are most likely related to a failure of earlier stapes surgery, II. an early-elective type mostly related to a persisting conductive hearing loss usually 12 to 60 months after the latest stapes or middle ear surgery, and III. a late-elective type 5 years or later after the latest stapes or middle ear surgery mostly related to acute-recurrent conductive hearing loss or slowly-progressive sensorineural hearing loss.

The acute type of stapes revision surgery usually depicts a kind of “rescue” surgery to prevent permanent loss of hearing and/or vestibular function [22]. Our retrospective cohort showed a mean increase in pure tone audiometry. The most common intraoperative findings were perilymph fistula as well as potentially toxic-irritative processes in the acute healing process such as granulation tissue which has been described as a cause of postoperative deafness after stapes surgery and may be an inflammatory foreign-body reaction caused by the prosthesis [28]. Therefore, we think that an acute revision usually consisting of a tympanoscopy and in most cases sealing of the oval window niche is justified when a severe acute hearing loss and vertigo occurs that is most likely linked to an earlier stapes surgery. To seal the oval window niche, fascia of the temporal muscle is one of the materials that is frequently used in stapes surgery [29]. In a third of the acute revision cases, explantation without implantation of a new stapes prosthesis was performed. Therefore, this surgical strategy was most likely in those acute cases. Nevertheless, any surgical strategy was accompanied by a conservative treatment regime usually consisting of high dose corticoids and i.v. antibiotics, in most cases of our cohort with ceftriaxone. Recommendations for an antibiotic therapy are based on principles of the perioperative management of middle ear surgery and do not appear to be mandatory based on the literature [30, 31]. In case of intraoperative risks such as tympanosclerosis or possible toxic-irritative processes in the middle ear after surgery, antibiotic therapy seems to be reasonable [32].

Early-elective stapes revision surgery was usually performed when a conductive hearing loss persisted after stapes surgery. This also explains intermediate time intervals between the prior and the revision stapes surgery because wound healing was awaited for a few months and, in several cases, a conservative treatment such as hearing aids was tried before a revision was conducted. In our cohort, scar tissue, tension phenomenon, and loose prostheses were disproportionately seen intraoperatively in those cases and, in most of those cases, scar tissue was removed and, in the majority of cases the stapes prosthesis was exchanged. Subacute stapes revision surgery reflects a smooth transition between the acute type and early-elective type of stapes revision surgery both regarding the indication and surgical strategies.

Late-elective stapes revision surgery was performed years after the prior stapes surgery, for various reasons including tinnitus, recurrent conductive hearing loss and progressive sensorineural hearing loss. Nevertheless, the most common intraoperative findings included incus erosions as well as loose and dislocated prostheses which is comparable to the data of Luryi et al. (incus erosions: 39.5% vs. 38%; loose/dislocated prosthesis: 66.9% vs. 81%) [33]. Interestingly, incus erosions and loose prostheses were also frequently seen in patients who had reported a slowly progressive hearing loss, even though a rather acute, recurrent hearing loss would be expected. Pathomechanisms discussed include pinching of the intra-osseous blood vessels by the stapes prosthesis, which may be too tight, or erosion due to scar traction [11, 21, 24]. Therefore, a careful removal of scar tissue belongs to the most common steps in stapes revision surgery which, in our experience, can sometimes solely restore the motility of the ossicle chain and reduce the conductive hearing loss.

Another common finding in stapes revision surgery is a bony obliteration of the perforated stapes foot plate. This finding has been linked to an incomplete footplate fenestration [34]. Alternatively, our experience from single cases suggests that stapes prostheses chosen too short might tend to extrude and enable the stapes footplate to re-obliterate over time. Moreover, fixation of the head of the malleus is a rare finding in stapes revision surgery that can lead to the aforementioned symptom of persistent conductive hearing loss, that may have been overseen in the prior surgery or slowly evolved after it. In our experience, it can be helpful to explant the stapes prosthesis before the fixation of the malleus is treated, to protect the inner ear. Frequently in these cases, fixation of the new prosthesis on the malleus (malleovestibulopexy) is performed. Although it is rare, both in cases with destructed or missing ossicles and in combined hearing loss active middle ear implants such as the vibrant soundbridge might be used in stapes revision surgery, e.g. as a “power stapes” attached to the long or more favorable to the short crus of the incus [35–38].

An important limitation of this study is the retrospective design. This is most importantly reflected in the data collection, both regarding potentially missing data points and regarding precision of the data, especially for tinnitus and vertigo where a differentiation of an objective or subjective finding was not possible in many cases. Unfortunately, even in some of the cases of acute deterioration of BC only unspecified “vertigo” was documented, beside a deterioration in subjective audiometry and e.g. a loud new tinnitus. Especially in cases with external prior stapes surgery, the history of hearing loss may be based on oral history if external tone audiometry was not digitalized or reported. If tone audiometry results were not digitalized for some of the early cases of the collective and also in a significant number of acute cases, standardized tone audiometry which is shown in the audiometric results was not available because symptoms occurred e.g. in the late evening, night or weekends and no standardized tone audiometry but e.g. fork testing or bedside tone audiometry was performed. In these cases, neither pre- nor postoperative audiometric data was presented.

Because of the variety of different preoperative findings and their heterogeneity of intraoperative findings, a selection of clinically relevant subcohorts was difficult. Since different prosthesis types were used and the number of cases for some types is limited, no exploratory subcohort analyses were conducted. Eventually, recurrent and persistent conductive hearing loss were selected to take a closer look at the audiological outcome, as it was done by Luryi et al. [33]. In our cohort and although we had fewer numbers in the subcohorts, we can confirm their finding that air–bone gap closure was lower in persistent rather than recurrent hearing loss [33]. Nevertheless, patients of both subcohorts can profit from stapes revision surgery which was also concluded by Luryi et al. and is especially seen in our relative outcome measures as described before [7, 33]. Regarding audiological outcome measures in stapes surgery, especially the relative hearing improvement shows that an overclosure phenomenon of the bone conduction might in particular be present in patients with a preoperatively reported persistent conductive hearing loss [7]. Especially in patients with recurrent or persistent conductive hearing loss, the test of Rinne can be used to guide decision-making regarding indication for revision stapes surgery with a negative finding more likely indicating an indication for revision stapes surgery and a positive test of Rinne likely not recommending revision stapes surgery but conservative treatment with hearing aids or in cases of severe sensorineural hearing loss cochlear implant surgery [7].

Considering the recent advances in this therapy, cochlear implants play an emerging role in the treatment of otosclerosis, especially in patients with severe combined or sensorineural hearing loss [39–41]. In this cohort, some patients that had been treated with stapes surgery eventually received a cochlear implant. In the rather small subcohort of this study, a priorly implanted stapes prosthesis was not removed during the cochlear implant surgery. In one case, acute deafness that occurred a few days after stapes revision surgery led to cochlear implant surgery. In cases like this, when a causative or accompanying labyrinthitis can be expected, a timely cochlear implant surgery should be provided before sclerotic changes of the inflammation obliterate the cochlea [42]. In our cases of far advanced otosclerosis, usually, round window insertion could be achieved, only rarely cochleostomy was needed and we did not see nonauditory stimulation of the facial nerve, so far, but also found satisfactory hearing results in all of those patients [43]. Based on our data and experiences, we therefore agree with Abdurehim et al. and Teaima et al. that patients with far advanced otosclerosis need to be counseled carefully because frequently both stapes revision surgery and cochlear implantation are reliable treatment options but cochlear implantation is considered a good alternative to stapes revision surgery, especially in cases with severe predominantly sensorineural hearing loss [44, 45].

## Conclusion

In this explorative study, we are able to categorize stapes revision surgery based on the time interval between prior stapes surgery and revision and link those three categories to intraoperative findings. In acute stapes revision surgery, acute substantial sensorineural hearing loss and usually also vertigo lead to revision. Early-elective stapes revision surgery was usually performed when a conductive hearing loss persisted after stapes surgery. Late-elective stapes revision surgery was performed years after the prior stapes surgery, e.g. because of recurrent conductive hearing loss and/or progressive sensorineural hearing loss. Patients are likely to profit from stapes revision surgery, even after multiple revisions. Patients with conductive hearing loss benefit from stapes revision surgery. However, patients with recurrent compared to persistent conductive hearing loss may profit more from stapes revision surgery. As various intraoperative findings can be found, stapes revision surgery is highly demanding for otologists to find proper surgical solutions, especially because surgeons are facing a black box situation until opening the middle ear after stapes surgery, kind of opening a “Kinder surprise egg”. In severe predominantly sensorineural hearing loss, cochlear implantation is a promising alternative to stapes revision surgery.
